# Bone-like structure by modified freeze casting

**DOI:** 10.1038/s41598-020-64757-z

**Published:** 2020-05-13

**Authors:** Gurdev Singh, S. Soundarapandian

**Affiliations:** Department of Mechanical Engineering, Indian Institute of Technology Madras, Chennai, Tamilnadu 600036 India

**Keywords:** Biomedical engineering, Implants

## Abstract

Freeze casting has emerged as one of the most promising manufacturing methods to fabricate porous scaffolds in recent years. This is due to various reasons which include a wide range of materials which can be used in this process, easiness of the process, etc. One of the major objectives of this work was to fabricate bone-like structure by using a modified freeze casting process. In this work, Hydroxyapatite and Tricalcium phosphate scaffolds with bone-like structure were fabricated by understanding and utilizing the basic physics of freeze casting. Thermal conductivity of the base plate is a crucial factor for obtaining controlled pore and porosity distribution in a porous scaffold. It was found that designing the base plate with variable thermal conductivity has led to the formation of bone-like structure. Porous scaffolds were quantitatively analyzed for pore size and porosity distribution at center and circumference. Porosity at circumference was observed to be approximately dropped by 55%, a similar trend was seen for pore size. Therefore, it was significant evidence that modified freeze casting has capable in fabricating bone-like structures with ease and good control. This will open many new applications of porous scaffolds in biomedical, energy devices, chemical catalyst and many more.

## Introduction

Mimicking the structure of a natural bone has always been the utmost priority in the field of science and engineering. Natural bone consists of bigger pores at the center as compared to circumference and the same trend follows for the porosity also. Freeze casting is a near-net shape manufacturing process to fabricate porous scaffold with ease. In this process, slurry of the main material is made with a freezing vehicle (water, camphene, etc.). The slurry is then frozen to certain conditions, followed by sublimation of freezing vehicle which leads to the formation of a porous scaffold. This scaffold has sintered at a temperature depending upon the thermal properties of the main material. The freezing step is where most of the scaffold properties are established. Solidification front velocity (${\rm{v}}({\rm{s}})$) plays an important role in deciding the pore morphologies of a porous scaffold during the freezing process. The solidification front velocity depends upon various factors such as thermal conductivity of solid layer (λ_S_), cooling liquid temperature (T_0_), thermal transition temperature (T_E_), latent heat of freezing vehicle (h_E_), thickness of solid layer (s) of freezing vehicle and density of slurry (ρ) but most importantly, the thermal conductivity (k) of base plate^1^

1 $${\bf{v}}({\bf{s}})=\frac{{{\boldsymbol{\lambda }}}_{{\bf{S}}}({{\bf{T}}}_{{\bf{E}}}-{{\bf{T}}}_{{\bf{0}}})}{{\boldsymbol{\rho }}{{\bf{h}}}_{{\bf{E}}}}{\left({\bf{s}}+\frac{{{\boldsymbol{\lambda }}}_{{\bf{S}}}}{{\bf{k}}}\right)}^{-{\bf{1}}}$$

It is clearly seen in Eq. 1 that the solidification front velocity is depending directly on the thermal conductivity of base material. Also, it is indicating that if the value of the thermal conductivity of the base plate is varying, it is giving a rise to different solidification velocities at different locations. The controlling of solidification velocity is directly leading to the control of pore size and porosity throughout the scaffold.

**Figure 1:**
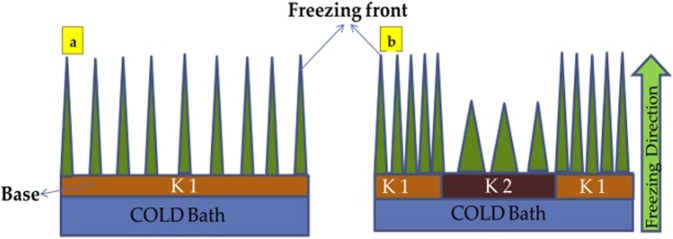
(**a**) Conventional freeze casting process with a base or cold finger of uniform thermal conductivity (K1) (**b**) modified freeze casting process with variable thermal conductivity base (K1 and K2), where K1 > K2.

Currently, freeze casting is capable only of fabricating porous scaffolds with almost similar pore size and porosity throughout the structure at given solid loading^2^. In freezing step of freeze casting process, a uniform thermal conductivity-based material is used as base plate. This is leading to uniform dendrite formation throughout the scaffold, specifically near to the cold end (Fig. 1a). In this work, freezing step of present freeze casting process was modified so as to achieve desired pore size and porosity in the scaffold. Base plate of variable thermal conductivities were used to achieve this. This modification in the base plate has influenced the formation of dendrites (Fig. 1b). Earlier, sequential freeze casting was used to fabricate porous scaffold with variable porosity^3^. But, one of the major drawbacks is that it is a two-step complex process. So, there is a requirement to fabricate porous scaffold by a simpler and effective approach. This work was aiming to obtain porous scaffold with variable porosity and pore size at the desired location with simple and effective manner. This approach has led to fabricate bone-like structures by freeze casting which was not possible before. In this work, the thermal conductivity of the base plate has been varied and its effect has been studied in terms of pore size and porosity of scaffold.

### Methodology

In the present work, methodology was divided into two sections. β-tricalcium phosphate (β-TCP) was used to verify the hypothesis and hydroxyapatite (HA) was to fabricate bone-like scaffold as structurally. Particles size of HA (20 nm) and β-TCP (10 μm) were used as primary materials. As received commercially available camphene was used as a freezing vehicle without any additive. For hypothesis testing, the samples of β-TCP with camphene with solid loading of 30 vol.% were prepared by using the magnetic stirrer at 60 °C for 3 h. In order to prove the concept of obtaining different pore sizes and porosities at a desired location of the scaffold, the thermal conductivity of the base plate or cold finger was varied. So, instead of keeping the base plate of uniform thermal conductivity, its thermal conductivity was kept as variable. Two combinations of the variable thermal conductive plates were used i.e. Polyethylene/Aluminum foil and Kapton tape/Aluminum foil. Aluminum foil has covered the circumference, whereas polyethylene/Kapton tape has covered the center. The prepared slurries were then transferred to polylactic acid (PLA) based mold (15 mm in diameter and 30 mm in height). β-TCP/camphene slurry was frozen at 5 °C with the base plate of various thermal insulators (Kapton tape and Polyethylene tape) combined with aluminum foil (Table 1). The configuration of the cold plate for conventional and modified freeze casting can be seen in Fig. 1. Samples were deep-frozen at −15 °C for 30 min in order to enhance the green strength before de-molding. In order to create a porous structure, samples were kept at room temperature for the sublimation of camphene for 24 h. After sublimation, scaffolds were then sintered using the muffle furnace at 1250 °C for 3 h with the heating and cooling rate of 10 °C/min.

**Table 1:** Thermal conductivities of various materials used as a cold base.

Property	Kapton Tape^5^ (K2.1)	Polyethylene Tape^6^ (K2.2)	Aluminum Foil^7^ (K1)
Coefficient of thermal conductivity (W/ (m.K))	0.12	0.33	235

Once the hypothesis was established, Kapton tape/Aluminum foil was used as the base material to fabricate HA scaffolds at different solid loadings i.e. 35 and 40 vol.%. To confirm the bone-like structure, scaffolds were characterized for pore size and porosity distribution at center and circumference, along with compressive strength.

## Results

### Variable thermal conductivity

#### Pore size

**Figure 2:**
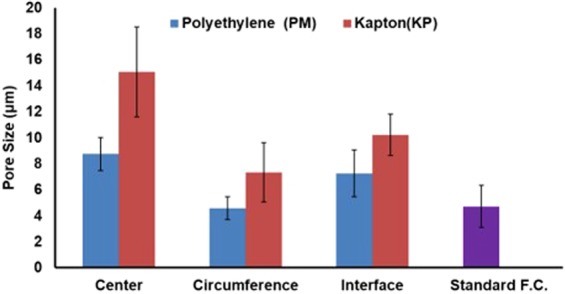
Comparison of pore sizes of scaffold fabricated with different thermally conductive materials and at various regions with respect to standard freeze casting.

It can be noted from Fig. 2 that there is a variation in pore at different locations of the β-TCP scaffold. At center, the pore size was observed to be greater than that at circumference for both cases of insulating material (Polyethylene (PM) and Kapton (KP) Tape). For PM as insulation, center, circumference, and interface has a pore size of 8.75 μm, 4.58 μm, and 7.26 μm, respectively. However, for KP as insulating material at the center, the pore size at the center, circumference, and interface was observed to be 15.06 μm, 7.33 μm, and 10.23 μm, respectively. Pores formed in both cases i.e. PM/Al and KP/Al, were higher than that of formed by standard freeze casting with uniform thermal conductive base.

#### Porosity

**Figure 3:**
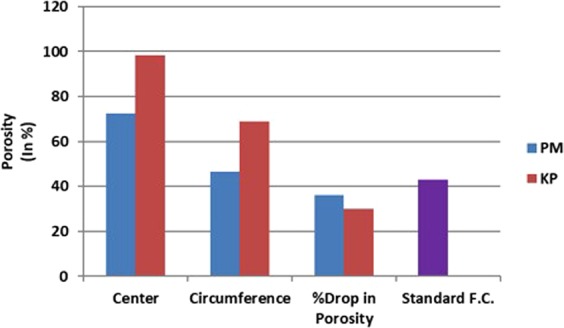
Porosity in various regions and different insulating materials.

Porosity is one of the important factors in deciding the biocompatibility of the scaffold. It can be clearly observed (Fig. 3) that thermal conductivity has clearly influenced the porosity level at various locations of the scaffold. The area of lower thermal conductivity (at center) has led to higher porosity than the area of higher thermal conductivity (Circumference) for both cases of insulations. Also, porosity in the case of KP was greater than that of PM at both center (KP: 78.88%; PM: 72.61%) and circumference (KP: 68.92%; PM: 46.42%) due to KP’s lower thermal conductivity. For both cases of thermal conductive materials, porosity of the scaffolds were greater than that of scaffold (Fig. 3) fabricated by standard freeze casting (43%).

### Variable solid loading

It was proved that variation of thermal conductivity along the base plate has resulted in better control on pore size and porosity at desired locations. Strengthening the concept further, solid loading has been varied by having a base plate of variable thermal conductivity (KP/Al foil) and HA (Nanoparticles) as the primary material.

### Pore size

**Figure 4:**
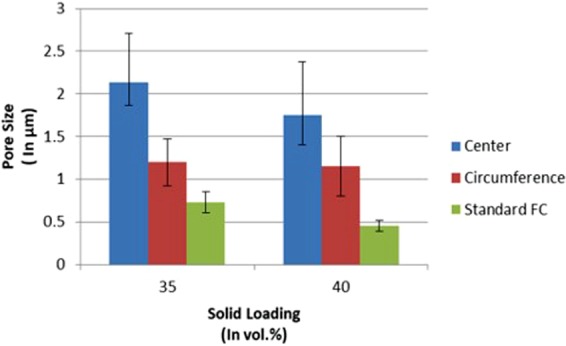
Comparison of pore sizes of scaffold fabricated at different solid loadings, regions and freeze casting methods.

**Figure 5:**
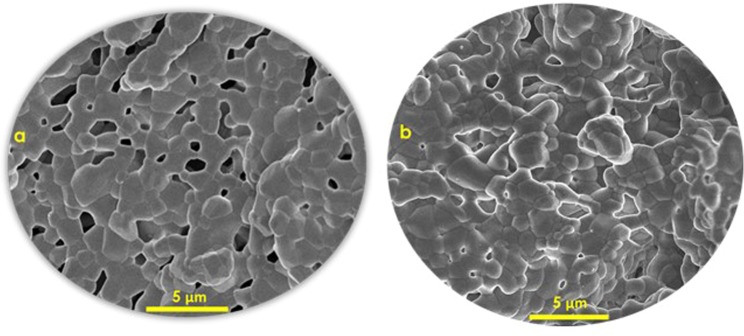
SEM image of a scaffold at given cross-section fabricated with (**a**) base of uniform thermal conductivity (**b**) base of variable thermal conductivity (Composite image by combining SEM images at circumference and center at the same cross-section and magnification factor (x5000)).

The same trend of bigger pores at the center as compared to circumference was observed at both solid loadings (35 vol.% and 40 vol.%) with a base plate of variable thermal conductivity (Figs. 4 and 5). The pore size at center and circumference for 35 vol.% solid loading was noted to be 2.14 μm and 1.2 μm, respectively. Similarly, for 40 vol.% solid loading pore size at center and circumference was observed to be 1.75 μm and 1.15 μm, respectively. Also, it can be noted that the average pore size of scaffolds fabricated at given solid loading with uniform thermal conductive base plate was lower as compared to the scaffolds fabricated by base plate of variable thermal conductivities (Fig. 4). Average pore sizes in case of scaffold fabricated by modified freeze casting at 35 vol.% and 40 vol.% were 1.7 μm and 1.54 μm, respectively. However in case of standard freeze casting, average pore sizes for solid loadings of 35 vol.% and 40 vol.% were 0.73 μm and 0.45 μm, respectively^4^. Also, it can be observed from Fig. 5 that pores at centers are bigger than pores at circumference. This indicates that varying the thermal conductivity has indeed influenced the formation of dendrites and hence, pore size at desired locations.

### Porosity

**Figure 6:**
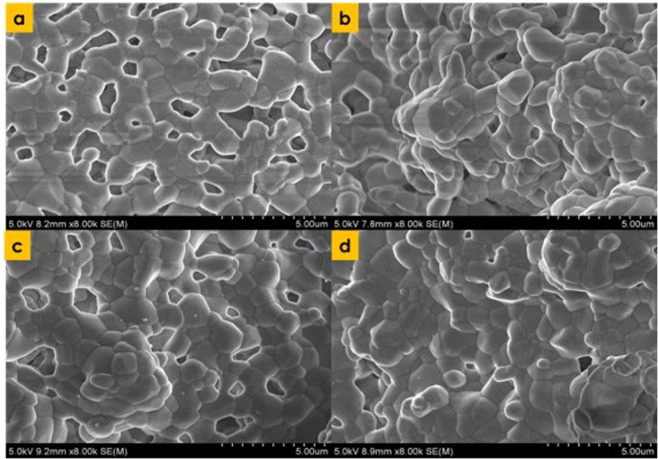
Pore microstructure at the solid loading of 35 vol.% (**a**) Center (**b**) Circumference and 40 vol.% c) Center d) Circumference.

Porosity at the center was greater than that at circumference for both cases of solid loadings (Fig. 5). At center and circumference, the porosity was observed to be 83.67% and 37.49% for the solid loading of 35 vol.%. And similarly, for 40 vol.% solid loading the porosity at center and circumference was 72.83% and 35.38%, respectively. The drop in porosity i.e. center to circumference was observed to be 55.19% and 51.42% for solid loading of 35 vol.% and 40 vol.%, respectively. Average porosities of the scaffolds fabricated by modified freeze casting were 60.58% and 54.11% for the scaffolds fabricated at 35 vol.% and 40 vol.%, respectively. Average porosities were 71.8% and 63.2% for the scaffolds fabricated by standard freeze casting^4^. It can be observed from Fig. 6 that pores are bigger at the circumference for both solid loading (35 vol.% and 40 vol.%) than that at center for HA scaffold.

### Compressive strength

**Figure 7:**
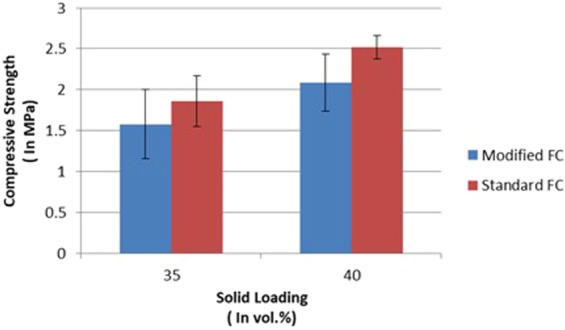
Comparison of compressive strength of the scaffolds fabricated at various solid loadings and freeze casting methods.

It’s not only the structures which are important but their mechanical behavior is also important in the case of load-bearing applications. In the case of bone, compressive strength is an important mechanical property which must be studied. The compressive strength of porous scaffolds by standard freeze casting and modified freeze casting were studied at various solid loadings. Average compressive strength in case of scaffold fabricated by modified freeze casting at 35 vol.% and 40 vol.% were 1.58 MPa and 2.09 MPa, respectively (Fig. 7). However, it was 1.86 MPa and 2.5 MPa for scaffolds fabricated at 35 vol.% and 40 vol.% by standard freeze casting^4^.

## Discussions

### Variable thermal conductivity

Also, it can be observed from Fig. 2 that pore sizes at different locations (Center, circumference and interface) for scaffold fabricated by keeping KP as insulation the pore sizes achieved were bigger as compared to pore sizes achieved by PM as insulation at a given location. This can be justified by the fact that KP has lower thermal conductivity than that of PM which allows camphene dendrite to grow more slowly and widely which results in large pores. Further, it was observed that pore size at the center for KP as insulating material was bigger than that of standard freeze casting process (Fig. 2). As explained earlier, lower thermal conductivity leads dendrites to grow larger which ultimately results in higher porosity.

### Variable solid loading

It was observed that at center pore size was bigger than that at circumference. This can be accounted by the fact that the location of the base plate where thermal conductivity is less, pore size tends to be bigger due to the proper growth of dendrites of camphene. Also, pore size has decreased with an increase in solid content as an increase in solid content hinders the growth of freezing vehicle during freezing step.

The difference in porosity at circumference and center is due to the difference in thermal conductivities at these areas of the base plate. Thermal conductivity of the base plate at the center is lesser than that of at circumference. This allows solidification front to move slow and spread which is responsible for the higher porosity at the center than that at the circumference. However, drop-in porosity for both cases of solid loading was similar, 55.19% and 51.42%, as at both solid loading same freezing and thermal conditions were used.

From Figs. 5 and 6, it can be visually observed that how porosities and pores are distributed throughout the scaffold for both cases of freeze casting methodologies i.e. standard freeze casting and modified freeze casting. It can be seen from Fig. 5 that in case of scaffold fabricated by standard freeze casting, almost similar sizes of pores are distributed throughout the scaffold. Similarly, porosity is homogeneously distributed in the case of scaffold fabricated by the standard freeze casting process. On the other hand, in case of the modified freeze casting process, pores and porosity are region-specific. In modified freeze casting, porosity and pores are distributed with greater control (Figs. 5 and 6). This has well established the fact that the porosity and pore size can be controlled by modifying the thermal conductivity of the base plate. That is, the center has higher porosity and bigger pores which indeed is the case of natural bone.

It was observed that at both solid loadings, standard freeze cast scaffolds had slightly better compressive strength as compared to modified freeze cast scaffolds (Fig. 7). This is due to the fact that porosity and pore of scaffold fabricated by modified freeze casting were greater which has led to lower compressive strength. Also, for both types of freeze casting the compressive strength at higher solid loading was better than at lower solid loading. It was already established that higher solid loading is responsible for higher compressive strength due to higher solid content^4^. Although, compressive strength of the scaffold was in the range of cancellous bone for both solid loading but it should be near to compact bone so that it can be used as bone implant. Relationship between average pore size, porosity, and compressive strength can be seen in Table 2 for the scaffolds fabricated by modified freeze casting.

**Table 2:** Summary of average pore size, porosity, and compressive strength of the scaffold fabricated by modified freeze casting.

Solid Loading (vol.%)	Pore size (µm)	Porosity (%)	Compressive Strength (MPa)
35	1.7	60.58	1.58
40	1.54	54.11	2.09

## Conclusions

Porous scaffolds with not only controlled porosity and pore size were successfully fabricated by modified freeze casting but also it was established that porosity and pore size are greater than that of scaffold fabricated by standard freeze casting method. Following are some conclusions from this work:

- It was observed that porosity in the case of scaffold prepared by this method was greater than that of prepared with a base of constant thermal conductivity
- Porosity and pore size were observed to greater for samples fabricated at 35 vol.% solid loading than that of at 40 vol.% solid loading
- Porosity and pore size of the scaffold fabricated with insulation method had larger porosity and pore size with respect to the standard freeze casting method
- It was proved in this work that porous scaffold of varying pore sizes can be fabricated by modified freeze casting method
- It can be concluded from this work that the thermal conductivity of the base material affects the microstructural and mechanical behavior of the porous scaffold fabricated by freeze casting

Modified freeze casting will be an important milestone in the evolution of freeze casting process. This will surely widen the scope and application of the freeze casting. These applications includes energy devices, better catalysts and most importantly in the field of biomedical industries. Bone implants with structure near to natural bone can be fabricated by modified freeze casting process.
